# Modelling eNvironment for Isoforms (MoNvIso): A general platform to predict structural determinants of protein isoforms in genetic diseases

**DOI:** 10.3389/fchem.2022.1059593

**Published:** 2023-01-09

**Authors:** Francesco Oliva, Francesco Musiani, Alejandro Giorgetti, Silvia De Rubeis, Oksana Sorokina, Douglas J. Armstrong, Paolo Carloni, Paolo Ruggerone

**Affiliations:** ^1^ Department of Physics, University of Cagliari, Monserrato (CA), Italy; ^2^ Institute of Neuroscience and Medicine INM-9, Institute for Advanced Simulations IAS-5, Forschungszentrum Jülich, Jülich, Germany; ^3^ Laboratory of Bioinorganic Chemistry, Department of Pharmacy and Biotechnology, University of Bologna, Bologna, Italy; ^4^ Department of Biotechnology, University of Verona, Verona, Italy; ^5^ Seaver Autism Center for Research and Treatment, Icahn School of Medicine at Mount Sinai, New York, NY, United States; ^6^ Department of Psychiatry, Icahn School of Medicine at Mount Sinai, New York, NY, United States; ^7^ The Mindich Child Health and Development Institute, Icahn School of Medicine at Mount Sinai, New York, NY, United States; ^8^ Friedman Brain Institute, Icahn School of Medicine at Mount Sinai, New York, NY, United States; ^9^ The School of Informatics, University of Edinburgh, Edinburgh, United Kingdom; ^10^ Simons Initiative for the Developing Brain, University of Edinburgh, Edinburgh, United Kingdom; ^11^ Department of Physics, RWTH Aachen University, Aachen, Germany; ^12^ JARA-Institute: Molecular Neuroscience and Neuroimaging, Institute for Neuroscience and Medicine INM-11/JARA-BRAIN Institute JBI-2, Forschungszentrum Jülich GmbH, Jülich, Germany

**Keywords:** isoform identification, mutations, molecular modelling, proteins, diseases

## Abstract

The seamless integration of human disease-related mutation data into protein structures is an essential component of any attempt to correctly assess the impact of the mutation. The key step preliminary to any structural modelling is the identification of the isoforms onto which mutations should be mapped due to there being several functionally different protein isoforms from the same gene. To handle large sets of data coming from omics techniques, this challenging task needs to be automatized. Here we present the MoNvIso (Modelling eNvironment for Isoforms) code, which identifies the most useful isoform for computational modelling, balancing the coverage of mutations of interest and the availability of templates to build a structural model of both the wild-type isoform and the related variants.

## 1 Introduction

The spatial and functional diversity of the 20,465 protein-coding genes (Howe et al., 20212021) (https://www.ensembl.org/) in the human genome is dramatically augmented through alternative splicing that results in an enormous number of potential protein isoforms. Exact numbers are not fully known but common estimates for total isoforms are in the 10X range (245,000 transcripts in https://www.ensembl.org/). Alternative splicing can result in isoforms with relatively subtle changes through to those that vary enormously in their structure, function, and subcellular spatial expression (Park et al., 2018).

Indeed, most functional (and dysfunctional) biochemical processes are affected by the expressed isoforms, which feature distinct functional roles. Examples of this complexity include the neuroligin and neurexin families, which perform synaptic regulatory functions that are surprisingly isoform specific (Markwick et al., 2007; Slabinski et al., 2007). This complexity may be increased by the addition of genetic variants, which can directly influence the protein structure and function of the isoform. Moreover, genetic variations can also affect the splice mechanisms and change the isoforms directly (Park et al., 2018), but this is not addressed in this study.

Further information, key to our understanding of genetic diseases, is the availability of three-dimensional structures of a protein. The structure of many human proteins is now available by accurate - yet time-consuming (Markwick et al., 2007; Slabinski et al., 2007) - experimental techniques (such as X-ray diffraction, NMR and electron microscopy (Murata and Wolf, 2018)). These accurate but demanding approaches are complemented by fast (and more approximate) computational predictions (Kuhlman and Bradley, 2019), including homology modelling (Kuhlman and Bradley, 2019) and deep learning techniques such as AlphaFold (AF) (Tunyasuvunakool et al., 2021), based on experimental structural information of evolutionarily related template protein(s) (Kuhlman and Bradley, 2019). Unfortunately, all these methods do not usually provide the isoforms most likely involved in the process of interest.

Here we present a computational platform that selects specifically the most useful isoform for molecular modelling and provides structural information, in the context of identified genetic variants. The presence of a variable number of protein isoforms makes it challenging to assign each mutation to a specific position in the protein sequence, which frequently hampers a reliable assessment of the impact of the genetic variations (including disease relevant mutations (Rees et al., 2010; Kato et al., 2018)) on an isoform suitable for molecular modelling. In other cases, a mutation is observed that is relevant to a specific isoform, but the databases reporting mutations related to a particular genetic disease usually lack a reference to the specific isoform.

Given a set of mutations at the protein expression level, our pipeline can correctly assign them to the corresponding isoforms at the protein level, providing important information that can be used for further investigations. The second key step of the determination of the isoform most useful for molecular modelling is achieved by combining the mutation-isoform map with the sequence coverage of available structural templates.

## 2 The MoNvIso (Modelling eNvironment for Isoforms) pipeline

**FIGURE 1:**
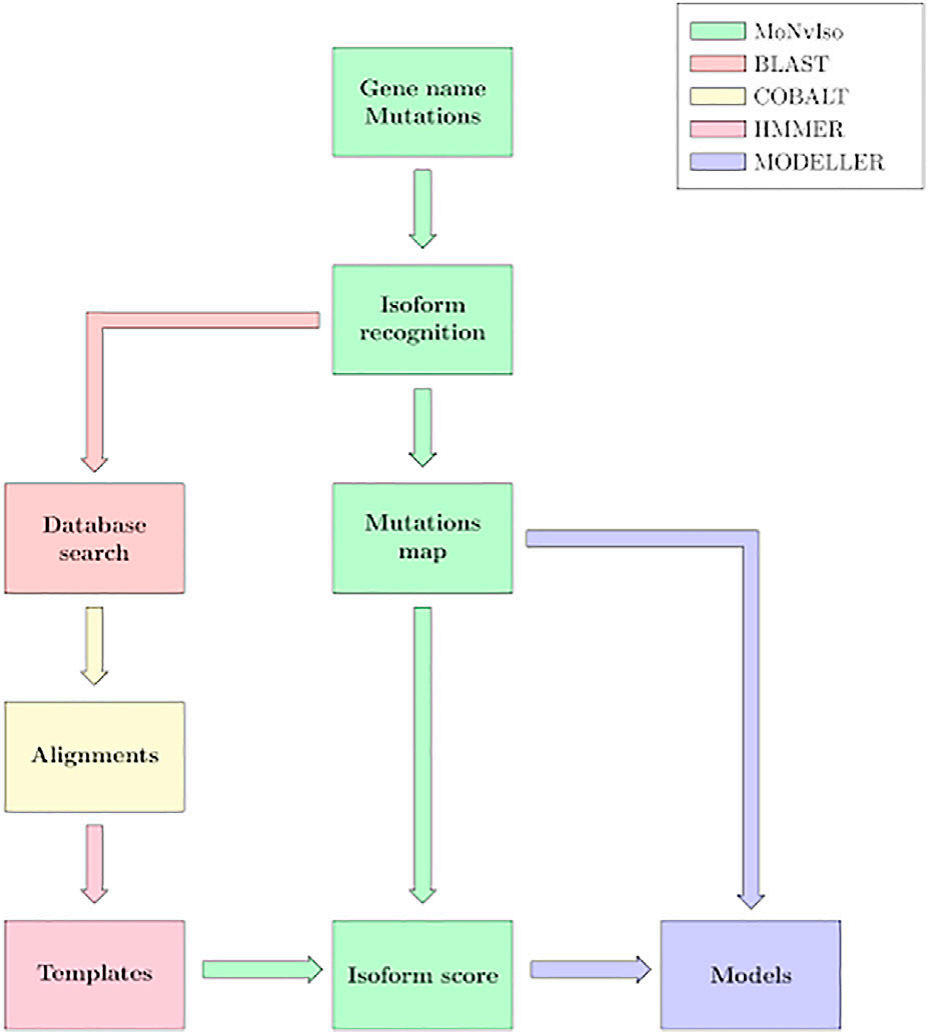
MoNvIso’s flowchart.

The general workflow of MoNvIso is summarised in Figure 1 and proceeds according to three steps described in more details in the next subsections:

1) Step 1: check of the gene names provided in the input file, identification of canonical and additional isoforms extracted from the Uniprot database. In the input file a list of the mutations of interest is also present.
2) Step 2: check of the modelling propensity and how properly mutations are mapped on the available isoforms. The availability of templates is supervised by MoNvIso, as well as the association of the mutations to the appropriate isoforms. MoNvIso highlights failures in this mapping procedure, i.e., when mutations cannot be mapped on any available isoforms.
3) Step 3: Building of the structural model of the identified proteins. Model of the wild-type (WT) forms and of their variants (selected by MoNvIso according to Step 2) are built if experimental structures are not already available for the selected isoforms.

The selection procedure is based on a function, named ***Selection***, (Step 2) that casts two contributions as follows:

Selection=w1⋅(Structural function)+w2⋅(Mutation function) (1)

The two terms, ***Structural function*** and ***Mutation function*** numerically translate the modelling propensity and the mapping of the mutations on the available isoforms to accomplish the two conditions. *w1* and *w2* are the weights of two terms. By default, *w1* = *w2* = 10 but they can be adjusted by the user. ***Structural function*** and ***Mutation function*** are described more in detail in the Subsection Step 2.

Collections of input and output files for the proteins KRAS and KDM5C are collected in example_p1. rar and example_p2. rar, which can be downloaded at https://github.com/MoNvIsoModeling/MoNvIso.

### 2.1 Step 1

MoNvIso checks the list of gene names and the set of point mutations provided by the user. The mutations can be indicated in the input file according to different formats: three-letters or single letter names for the amino acids. Additionally, spaces and tabs are also accepted to simplify the creation of the list by the user. Every gene name is searched against the Uniprot (Bateman et al., 2021) database, the results are extracted from two files, namely *uniprot_sprot.fasta*, which contains the aminoacidic sequence of the canonical isoforms according to the classification of Uniprot, and *uniprot_sprot_varsplic.fasta* collecting the sequences of the remaining isoforms obtained from Uniprot (see Supplementary Figure S1 for the list of folders and files created by MoNvIso) .

### 2.2 Step 2

MoNvIso then performs an analysis on each isoform extracted from the Uniprot entry (see Step 1) based on two functions: 1) checking the modelling propensity and 2) mapping of the mutations. A score is associated with each function and the combination of the two is used to select the isoform most suitable to be modelled. Independently on the chosen isoform to be modelled, the information on the mapped mutations onto all the isoforms is provided by MoNvIso. In detail:

#### 2.2.1 Checking the modelling propensity.

Each isoform is then processed according to a standard procedure: A search for homologous sequences is performed using BLAST API (Altschul et al., 1990), which allows users to submit BLAST searches for processing through cloud service provider(s) using HTTPS; and a multi sequence alignment (MSA) is generated using COBALT (Papadopoulos and Agarwala, 2007). Subsequently, based on the MSA, the hmmsearch function of HMMER (version 3.3.2 http://hmmer.org/) uses the HMM (Hidden Markov Model) (Baum and Petrie, 1966) to find relevant templates in the PDB. The 10 most similar sequences for the identified PDB structures are downloaded and the chains necessary for the homology modelling are extracted as separate files. The extracted structures are cleaned from water molecules, ligands, disordered atoms, and non-standard residues, then aligned to the MSA and are made available to the user in a folder (see Supplementary Figure S1).

The resulting structures are ranked by resolution and sequence identity to find the most appropriate templates, thus excluding crystals with poor resolution or with sequences that are very different from the original query (see Section Limitations). The default values of the sequence identity and resolution thresholds are 25% and 4.5 Å, respectively. However, the thresholds can be modified by the user. A further selection criterion is applied by calculating the coverage of the input sequence by the sequences of the templates. To this aim, MoNvIso identifies the minimum number of templates necessary to model the highest percentage of the target sequence. For a given target sequence (for example, Isoform 1 = ADRRVLTY) and the set of templates identified as described above (for example, Template A: AD, Template B: AD, Template C: RRVLT, Template D: DRR), MoNvIso proceeds as follows:

1) Sorting of the templates according to the covered lengths, in our case Templates A, B, D, C;
2) Checking if the given sequence is covered by more than one template or by a combination of templates. In our case, Templates A and B cover the same portion;
3) If a single template covers the target, then this template is considered (which is not the case of our example);
4) If the target is covered either by a longer template or by a combination of other templates (with at least one covering extra portions of the protein), the proper selection is considered. In our example, this is accomplished by the combination of Templates A and C, being the choice between Templates A and B only dictated by the alphabetical order.

The described procedure is applied by MoNvIso to entire sequences or portions of them and to all the possible additional isoforms (our example deals with a second isoform, Isoform 2 = ADRKVLTY). Note that information about covered sections and associated templates are stored in the *covered_intervals* file produced by MoNvIso.

Starting from the above description, the term ***Structural function*** in Eq. 1, accounts for the availability of crystallographic data defined as the number of amino acids (AAs) that are covered by a template (or a combination of templates) over the total number of AAs constituting the isoform

$$Structural\;function=\frac{\left(Covered\;AA\right)}{\left(Total\;AA\right)}$$ (2)

In the above example, for Isoform 1 we have ***Total AA*** = 8 and ***Covered AA*** = 7, resulting in a ***Structural function*** = 0.875, while for Isoform 2 the values of ***Covered AA*** and ***Structural function*** are 6 and 0.750, respectively.

#### 2.2.2 Mapping of the mutations

The second term of Eq. 1, ***Mutation Function***, considers the entire list of mutations provided for the considered gene, thus pinpointing to the isoform most suitable for homology modelling. Our program maps all mutations onto the appropriate isoform and increases by one the numerator, ***Mutating AA that can be modelled***, if the mutated residue can be correctly located in the isoform sequence. The contribution of matched mutations to the selection function is evaluated as follows:

$$Mutation\;function=\frac{\left(Mutating\;AA\;that\;can\;be\;modelled\right)}{\left(Mutating\;AA\;found\;in\;at\;least\;1\;isoform\right)}$$ (3)

According to our example, for the three mutations T2A, R3A, R4L, MoNvIso highlights that the first mutation T2A is not mapped on the two present isoforms, while it evaluates ***Mutating AA that can be modelled*** equal to two and one for Isoforms 1 and 2, respectively. ***Mutating AA found in at least one isoform*** is two for both isoforms, ***Mutation function*** (Isoform 1) = 1, and ***Mutation function*** (Isoform 2) = 0.5.

For each gene and each isoform, the resulting ***Selections*** are reported in the *report.* log file. Moreover, this file contains a report on all mutations inserted in the input file, that is, i) the mapped mutations, ii) on which isoform they were mapped and iii) mutations not associated with any isoforms, together with iv) the isoform most suitable to be modelled (see Supplementary Figure S2). In our example, the selected isoform to be modelled is Isoform 1 with ***Selection*** = 18.75.

### 2.3 Step 3

Structural models for the selected isoform in its WT form and in all the variant(s) associated with the properly mapped mutation(s) are then created by using the MODELLER program (Webb and Sali, 2016) based on the sequence alignment obtained in the previous step. Regions not covered by the templates are not considered. The models are then ranked by the DOPE score (Shen and Sali, 2006), and MoNvIso yields the top ranked one (the list of all the models with their DOPE score is in the file MYOUT. dat, see SI for the list of all the files generated by MoNvIso and their location). The modelling of the variants is then performed by taking the MODELLER input file containing the WT sequences of the templates and replacing the mutated AAs in the sequence. MODELLER is then run again to produce the model of the variant(s). This can be useful for mapping the position of mutations on a three-dimensional structure, allowing the study not only of the mutated residue but also of the amino acids in its vicinity and with which the mutated residue may be in contact.

## 3 Strengths

Our pipeline exploits a series of tools tailored to manage large sets of proteins. Useful information is provided at each step of the run so that decisions taken by the pipeline can be audited. In the case of a failure of the pipeline to provide a satisfactory structural model, the file *report. csv* traces the mutations on all the isoforms and provides an easy way to identify the isoform mapping the largest number of mutations. The previously mentioned *report*. *log* file is also important. This file contains all the data that would otherwise have to be manually collected such as the number of isoforms for a gene, the location of the mutations, which mutations cannot be mapped on any known isoform and finally the values of the selection functions. These data can provide a useful starting point if the user needs to manually model the protein. For example, the user, upon data retrieval, can also decide if another isoform should be prioritised because of a mutation of particular interest not present in the isoform selected by the program. Regarding the modelling part of the protocol, the final alignments, the used templates with detailed information on the selection process as well as the coverage are made available to the user, as specified thoroughly in Section 2. Although the process of building the variants can be time consuming if many of them need to be built, this part is fully automated. In most of the tested cases the models built showed a high quality and can be used for further studies (see Section Results). Thus, our pipeline reduces the time necessary to model a large number of proteins by automating the slowest parts of the process including the search for isoforms, the mapping of mutations, the search for crystallographic data to use as templates and the building of the alignments.

## 4 Limitations

As with any modelling study, also our method presents limitations. MoNvIso does not model the parts of the protein that are not covered by templates. The solution implemented in the program is the modelling of the single domains, although this implies the uncertainty on reciprocal orientations of the domains. An additional drawback is the possible presence of several small portions that can be modelled but are interspersed by regions not covered by templates. In some cases, the search for templates with HMMER does not return any result (depends on HMMER’s servers). When several successive searches for homologues are queued on BLAST, a slowdown of the runs may occur. Multiple point mutations coexisting on the same proteins are not modelled by MoNvIso concurrently. Rather, MoNvIso provides a series of structural models of single amino acid variants for pairwise comparison. Finally, MoNvIso selects the most useful isoform based on available structural data and mutation coverage but there is no guarantee this is the most functionally relevant one in every case.

## 5 Case studies

We tested MoNvIso on a set of 70 proteins. A corresponding 257 human isoforms were extracted from the Uniprot database and relative mutations obtained from the relative Uniprot webpage, with a maximum cap of five mutations per protein. The genes and mutations considered are listed in the file *mutations.txt* provided in Supporting Materials. For all selected proteins MoNvIso was able to produce the alignments and to map the mutations onto the identified isoform. It successfully located, retrieved, and edited the templates to generate the WT structural models as well as the variants, when the identified mutations were in the modelled portions.

Out of the 70 proteins we modelled, 53 WT models could be compared against equivalent ones available in the AF database (DB) (https://alphafold.ebi.ac.uk/). This was done by extracting from the AF model the part of the sequence that we modelled and performing an RMSD analysis on the Cα.

For the remaining 17 proteins (BCL11A, CACNA1B, CAMKK1, CAMKK2, DNMT1, FMR1, GABRB3, GRIK2, GRM5, PLXNB1, SCN2A, SLC17A8, SNAP25, STX1A, SYN1, SYT1, TAF1), such comparison was not feasible because the isoform selected by MoNvIso was not the canonical one as considered by AF and was not sufficiently similar for direct comparison, i.e. the number of Cα was different. For a further 13 proteins out of 70 we modelled an isoform different from the canonical sequence but the RMSD comparison with the AF models was possible because the changes were localised in region not covered by templates.

Thus, for a total of 30 proteins out of 70 mutations are best modelled on non-canonical isoforms. The results of the comparison are presented in Supplementary Table S1 together with the amount of residue for which AF has a high or very high confidence (pLDDT score >70) about their position. The genes are ordered from the one with lowest RMSD value to the highest. According to Supplementary Table S1, 44 out of 57 (77%) models present an RMSD below 20 Å, and a visual inspection reinforces the validity of our results, since the larger RMSD values in this group are mainly due to small, disordered loops. In the group of models with RMSD above 20 there are subunits assuming different orientations in both MoNvIso and AF structures. When comparing the number of AA with a high or, very high, confidence score, we see that in most of our results (46 out of 57), the modelled portion retains at least 50% of these residues.

**FIGURE 2:**
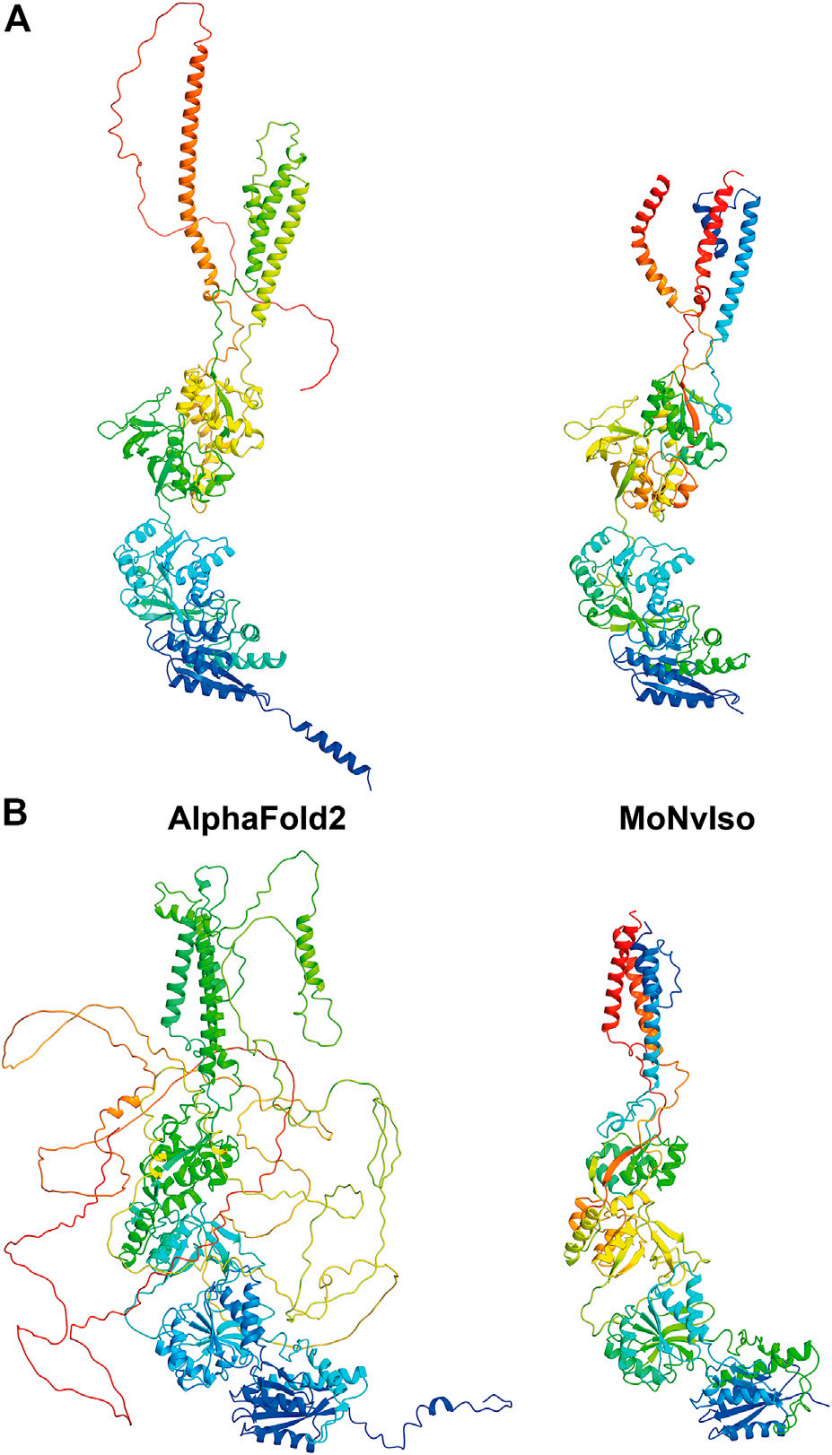
Comparison between the ribbon representations of GRIN1 **(A)** and GRIN2B **(B)** model structures generated with AF (left panels) and MoNvIso (right panels). The ribbons are colored from blue to red going from the N- to the C-terminal.

As an example, we show two structures in Figure 2: the proteins GRIN1 (Glutamate receptor ionotropic, NMDA one; also known as GluN1; Uniprot #Q05586) and GRIN2B (Glutamate receptor ionotropic, NMDA one; also known as GluN2B; Uniprot #Q13224). These two transmembrane proteins are subunits of the N-methyl-d-aspartate (NMDA) glutamate receptor complex, which contribute to excitatory transmission in the brain. In the first case both AF and MoNvIso produce similar results that differ only in the domains for which no templates are available, but still modelled by AF. Examples of these domains are the C-terminal part, starting from K866 to S938 and the N-terminal helix (residues M1 to D23) that are modelled by AF and not by MoNvIso (see top left and bottom right in Figure 2A, respectively). These two portions of the sequence are not considered by MoNvIso (see Step 3) since there are no available templates to correctly model them, but AF does attempt to model the whole chain. This leads to portions of the model with low or very low confidence scores (calculated by AF), and which corresponds to a pLDDT between 0 and 70, meaning that those parts of the model are generally unreliable.

The results for GRIN2B (see Figure 2B) demonstrate the differences between AF and MoNvIso predictions. AF successfully models the N-terminal part of the protein but fails to correctly build the trans and intra-membrane domains, which are then added as loops twisted around the correctly modelled section of the protein. Once again, the portions that are missing from the PDB database are poorly modelled. Since AF has been trained on the PDB dataset (Tunyasuvunakool et al., 2021), it still relies on available crystallographic data to correctly model structures. Thus, transmembrane domains such as those of GRIN2B, which are underrepresented in that training set because of the scarcity of experimentally determined structures of transmembrane proteins and their complexes (Kermani, 2021), may fail to be correctly built. In turn, MoNvIso automatically recognises the parts of the protein that can be modelled with confidence. As a result, MoNvIso cuts out of the sequence the extra AAs that cannot be modelled, producing a model ready to be used for further analysis.

## 6 Conclusion

Dissecting the impact of point mutations in the function of a protein are often hindered by a lack of an appropriate mapping of the mutation onto the correct isoform of a protein, of the identification of isoform(s) useful for molecular modelling, and of the associated building of a reliable structure. This knowledge is important because different isoforms of proteins can have widely differing functional roles and spatio-temporal expression profiles. As genomic variants associated with human traits and/or disease are being discovered at an increasing rate, approaches to link them to isoforms and find reliable structural models are urgently needed. MoNvIso addresses these two aspects: mapping a set of point mutations (provided by the user) on known isoforms, along with selecting the isoform most suitable to be modelled. The prediction of the structural models for the WT isoforms and their variants is automated, making MoNvIso appropriate for high-throughput investigations. Although several platforms to provide accurate structures of a protein are available and routinely used (Yang et al., 2014; Webb and Sali, 2016; Waterhouse et al., 2018), surprisingly few of them can be implemented in a pipeline (Webb and Sali, 2016) to automate the modelling of multiple different proteins. Therefore, our protocol combines this final step with the key preliminary assessment of the isoform mapping correctly the mutation of interest. Importantly, all steps of our protocol yield results that can be used at different stages by the user: the identification of specific isoforms containing residues involved in selected mutations is *per se* a remarkable clue for genetic assessment of the impact of isoforms, especially by handling a large number of proteins and point mutations; the set of the templates eventually identified by MoNvIso with the section of the target protein covered by them are made available to the user; finally, the structural predictions represent a valuable starting point for additional refinements and investigations, such as molecular dynamics simulations (Raval et al., 2012; Hollingsworth and Dror, 2018; Lazim et al., 20202020; Miller and Phillips, 2021; Itoh and Okumura, 2022), hot spots evaluation (Murakami et al., 2017; Liu et al., 20182018; Rosell and Fernández-Recio, 2018; Rosensweig et al., 2018), protein-protein docking (Kangueane and Nilofer, 2018; van Noort et al., 2021) and more (Poelwijk et al., 2016; Rivoire et al., 2016; Salinas and Ranganathan, 2018). Finally, note that for isoforms without good quality-templates, users could choose to use predicted structures such as those provided by AF and RosettaFold (Baek et al., 2021) or other modelling packages and/or protocols to build their own structural models using the isoform(s) correctly associated with the selected point mutations.

The test of MoNvIso on a set of proteins and the comparison with the results of AF confirms the validity of our approach. Additionally, our computational protocol can be easily inserted in a pipeline suitable to perform extensive campaigns of investigation on protein-protein interactions. MoNvIso is particularly useful to evaluate the availability of templates for large sets of proteins and automatically selecting the isoform most suitable to be modelled containing the point mutations of interest. MoNvIso is freely available and can be downloaded from GitHub at the following link: https://github.com/MoNvIsoModeling/MoNvIso, implemented in Python 3.8 and tested on version 3.0, 3.7 and 3.9 and supported on Linux.

## Key points

1) We have developed a computational protocol to map mutations on appropriate isoforms of protein.
2) The protocol identifies the available templates on which mutations can be located.
3) Ranking of the isoforms based on the number of located mutations and the template coverage.
4) Structural models are built for the WT and mutated isoforms if reliable templates are available.

## Data Availability

The datasets presented in this study can be found in online repositories. The names of the repository/repositories and accession number(s) can be found below: https://github.com/MoNvIsoModeling/MoNvIso.
